# Serotonin Toxicity Precipitated by Tramadol in the Setting of Polypharmacy: A Case of Serotonin Syndrome

**DOI:** 10.7759/cureus.20059

**Published:** 2021-11-30

**Authors:** Ariel Ruiz de Villa, Tyler Jones, Amina Lleshi, Monica Macahuachi, Katie Lamar, Yvette Bazikian

**Affiliations:** 1 Internal Medicine, University of Central Florida College of Medicine, Orlando, USA; 2 Internal Medicine, HCA Healthcare North Florida Regional Medical Center, Gainesville, USA; 3 Internal Medicine, Nova Southeastern University, Davie, USA

**Keywords:** serotonin syndrome, polypharmacy, tramadol, toxicity, serotonin

## Abstract

Serotonin syndrome (SS), a potentially life-threatening condition, typically occurs due to polypharmacy and interaction with multiple serotonergic agents. The case presented here is based on a serotonin syndrome (SS) diagnosis, precipitated by newly prescribed tramadol in conjunction with previously prescribed serotonergic medications. A 79-year-old woman receiving combined citalopram and trazodone for major depressive disorder alongside oxycodone for chronic pain developed generalized weakness, tremors, altered mentation, episodic auditory and visual hallucinations, fever, tachypnea, tachycardia, and diaphoresis a few days after tramadol was prescribed for pain. On clinical examination to medication reconciliation, and ruling out other causes of altered mental status, it became evident that the addition of tramadol had resulted in acute serotonin toxicity. SS is important to recognize because many healthcare providers encounter it during their careers. This diagnosis is essential to include in the differential diagnosis, especially when a medication not often associated with serotonin, like opiates, is the culprit.

## Introduction

Serotonin is an important neurotransmitter in the body and is involved in many processes, including mood, sleep-wakefulness cycles, and thermoregulation alongside emotional and food behaviors. Serotonin syndrome (SS), a potentially life-threatening condition, typically occurs due to polypharmacy and interaction with multiple serotonergic agents [1,2]. The resulting excessive serotonergic agonism in central and peripheral neurological receptors leads to autonomic dysfunction, neuromuscular excitation and altered mentation. The incidence is unknown, though the true number of cases is very likely to be much higher than the number reported, considering a majority of physicians are unaware of it being a clinical diagnosis [3,4]. Regardless, the incidence of cases is increasing in recent times. The clinical course is rapid and can be quite severe, with an onset of symptoms within six hours of medication initiation [4]. Sixty percent of patients recover 24 hours after treatment initiation [2]. This case provides a unique opportunity to observe an opiate, tramadol, precipitating SS in concomitance with oxycodone, citalopram, and trazodone.

## Case presentation

The patient was a 79-year-old Caucasian female with a past medical history of non-ischemic non-alcoholic cardiomyopathy, heart failure with reduced ejection fraction (30%-35%), major depressive disorder, diabetes mellitus type 2, chronic pain secondary to non-Hodgkin’s lymphoma status post-chemotherapy, diabetic neuropathy, arthritis, hypothyroidism, hypertension, hyperlipidemia, and obesity who presented to the emergency department with generalized weakness and worsening tremors with an onset of over a week at home that involved the upper and lower extremities bilaterally.

The patient reported severe tremors with an inability to hold on to objects in her hand, frequently causing her to drop things. She reported that the tremors intensified when standing from a sitting position, which had caused a fall with head-strike but no loss of consciousness. As per the patient’s husband, she had been having episodic visual and auditory hallucinations with associated palpitations and diaphoresis. Additionally, it was reported that the patient’s demeanor and mood had changed.

As per family, the patient had been compliant with all prescribed medications from her primary and pain control doctor as scheduled with the aid of husband and daughter. Table 1 depicts the medical reconciliation obtained during admission. Of note, a week prior to admission, the patient had been started on tramadol to supplement her pain regimen.

**Table 1:** Patient's medications

Medications			
Oxycodone 20 mg every 12 hours	Citalopram 20 mg daily	Trazodone 100 mg bedtime	Tramadol 50 mg twice daily
Gabapentin 600 mcg every 8 hours	Propranolol 20 mg twice daily	Metformin 500 mg twice daily	Levothyroxine 125 mcg daily
Lisinopril 40 mg daily	Carvedilol 6.25 mg twice daily	Furosemide 40 mg daily as needed	Aspirin 81 mg daily
Cholecalciferol 1000 units daily	Magnesium 250 mg daily	Ubidecarenone 30 mg daily	Elderberry 1 tab daily

Her vital signs were as follows: temperature 100.9°F, pulse 77 beats per minute, respiratory rate 19 breaths per minute, blood pressure 131/62 mmHg with the mean arterial pressure of 85 mmHg, and 92% saturation on room air.

Physical examination was significant for altered mentation, disorientation to self and situation, resting and intention tremors, diaphoresis, fever, episodic tachycardias and tachypnea, labored breathing and bilateral rales, 3+ pitting edema of the extremities, and new decreased sensation in her left foot up to her ankle. The patient did not participate appropriately during the initial physical exam due to altered mentation. Laboratory results showed a grossly normal complete blood count with the exception of an elevated white blood cell count of 13,200/mm^3^ with an increase in the number of immature cells. Chemistry results were abnormal for a creatinine level of 1.4 mg/dL and blood urea nitrogen level of 35 mg/dL, potassium level of 5.5 mmol/L and a lactic acid level of 1.9 mg/dL. Her B-type natriuretic peptide level was 3970 pg/mL with negative troponins. Thyroid studies were normal, with no previous baseline labs available. Chest x-ray demonstrated pulmonary vascular congestion and CT brain was negative for any acute pathology. Urinalysis and blood cultures were collected.

She was admitted to the hospital with a presumptive diagnosis of metabolic encephalopathy in the setting of heart failure exacerbation alongside sepsis secondary to community-acquired pneumonia, as she met the systemic inflammatory response syndrome (SIRS) criteria. She was treated empirically with ceftriaxone and doxycycline.

Given the extensive pharmacological regimen with the addition of a new medication around the same time of the onset of symptoms and with the classical triad of autonomic dysfunction, neuromuscular excitation and altered mentation, serotonin toxicity was suspected. Oxycodone and trazodone were held and tramadol immediately discontinued resulting in clinical improvement within 24 hours. The patient was alert, oriented and tremors had subsided the following day. Urinalysis and blood cultures were negative for infection. The patient was discharged after an additional day of observation with a new regimen of medications and instructions to follow up with her primary care physician. Both patient and family education was also provided.

## Discussion

SS is an underdiagnosed condition and the diagnosis requires a high degree of clinical suspicion [2] .There is no specific test to distinguish SS from its counterparts, such as alcohol withdrawal, metabolic and viral encephalopathy, neuroleptic malignant syndrome, anticholinergic toxicity and intoxication from sympathomimetic agents among others. Diagnosis by a medical toxicologist or a medical professional is the gold standard. This approach is often not feasible, depending on the setting and availability of such a specialist. Additionally, patients can have a wide range of presentations from mild symptoms to a life-threatening syndrome [4]. The clinical triad that characterizes SS includes autonomic hyperactivity, mental status changes, and neuromuscular abnormalities. Patients with mental status changes often exhibit disorientation, delirium, and are easily startled. Those with autonomic hyperactivity may experience diaphoresis, tachycardia, exaggerated changes in blood pressure, vomiting, and diarrhea. Neuromuscular abnormalities may include myoclonus and hyperreflexia, rigidity that may mask clonus and hyperreflexia, as well as a positive Babinski sign. These abnormalities are normally more prevalent in the lower extremities, and with respect to possible complications of SS, we may see rhabdomyolysis with subsequent renal failure and myoglobinuria, acute respiratory distress syndrome, and disseminated intravascular coagulation. Table 2 outlines the spectrum of symptoms of serotonergic toxicity.

**Table 2:** Spectrum of symptoms of serotonergic toxicity Source: Ref. [5]

Severity	Neuromuscular excitation	Altered mental status	Autonomic dysfunction
Mild	Hyperreflexia, tremor, myoclonus	Anxiety, restlessness, insomnia	Diaphoresis, mydriasis, tachycardia
Moderate	Opsoclonus, spontaneous or inducible clonus	Agitation	Hypertension, hyperthermia (<104°F), diarrhea, nausea, vomiting
Severe	Rigidity, respiratory failure, tonic-clonic seizure	Coma, delirium, confusion	Dynamic blood pressure, severe hyperthermia (>104°F)

Patients typically experience symptoms within six hours of administration of the offending agent. Although the timeline of onset of symptoms in our case is unclear, we suspect it was soon after the addition of tramadol to her medications. She was not hospitalized until seven days after beginning her new medication. This patient was already taking three drugs that put her at risk of serotonin syndrome (citalopram, trazodone and oxycodone). With a presentation consisting of fever and impaired coordination, altered mentation, and tremors, this would be considered a mild presentation of the syndrome [4]. Table 3 lists various classes of medications that can lead to a serotonin syndrome, since all of them increase the level or action of serotonin as a neurotransmitter.

**Table 3:** Drug mechanisms associated with serotonin syndrome Source: Ref. [5] MAOI, monoamine oxidase inhibitor; SSRI, selective serotonin reuptake inhibitor; SNRI, serotonin-norepinephrine reuptake inhibitor

Mechanisms	Agents
Serotonin receptor agonist	Buspirone, fentanyl, triptans, lysergic acid diethylamide, ergotamine
Decreased serotonin metabolism	MAOIs (e.g. phenelzine, selegiline, and isocarboxazine), antibiotics (e.g. linezolid), methylene blue
Increased serotonin release	Amphetamines, cocaine, methylenedioxymethamphetamine, mirtazapine
Increased serotonin production	Tryptophan
Decreased serotonin reuptake	SSRIs (e.g. fluoxetine, citalopram, and sertraline), SNRIs (e.g. venlafaxine and duloxetine), opioids (e.g. tramadol, meperidine, and dextromethorphan), antiemetics (e.g. ondansetron and metoclopramide), antiepileptics (e.g. carbamazepine and valproic acid), St. John’s wort cyclic antidepressants (e.g. amitriptyline and nortriptyline)
CYP2D6 and CYP3A4 inhibitors	Antiretrovirals (e.g. ritonavir), antibiotics (e.g. ciprofloxacin and erythromycin), antifungals (e.g. fluconazole)

Laboratory tests are not necessary for the diagnosis of SS, as they are not always abnormal. However, some associations are worth mentioning. Elevated total creatine phosphokinase, leukocyte count, and transaminase levels as well as lower bicarbonate levels are typical of SS [2].

Our patient's labs demonstrated an elevated leukocyte count of 13,200/mm^3^, normal transaminase levels of aspartate aminotransferase (AST) 19 units/L and alanine aminotransferase (ALT) 22 units/L, and a high-normal bicarbonate level of 29 mEq/L. Only the white blood cell count correlated with the typical findings.

The cessation of serotonergic agents and supportive measures are the mainstay of treatment. Intravenous fluids (lactated ringers or normal saline) are also administered in order to maintain the diuresis to 50-100 mL/h and to prevent myoglobinuria [2]. Oxygen saturation should be maintained above 93% [4]. Benzodiazepines may be used if the patient has a significant amount of anxiety. Cyproheptadine, chlorpromazine, and propranolol have all been reported as adjunct treatment [2]. Cyproheptadine prevents hyperthermia, the lethality of the syndrome, and can provide symptomatic relief, but it does not shorten the time course of the syndrome [2,5].

The utility of propranolol has been questioned by our team due to concerns regarding the masking of symptoms, tachycardia and hypertension, and may make it difficult to gauge clinical improvement. This patient was on propranolol before the syndrome was triggered. This raises the question, as to whether propranolol would be a useful adjunct in the treatment of SS. Further research is needed in regard to the utility of propranolol as part of treatment for SS.

Pharmacology

The washout period for selective serotonin reuptake inhibitors (SSRIs) may be prolonged in those with liver or kidney disease, as well as in older patients. SSRIs are metabolized via the cytochrome P450 enzyme in the liver. Not only that, SSRIs inhibit CYP2D6, the enzyme that metabolizes tramadol. The active metabolite of tramadol is O-desmethyltramadol, which exerts its effects by binding to μ-opiate receptors. Tramadol also acts by inhibiting the reuptake of serotonin and norepinephrine, modulating descending neuronal inhibitory pathways [6]. Similar to SSRIs, patients with renal failure, liver cirrhosis, and those who are older (>75 years old) require lower doses of tramadol. These patients accumulate higher concentrations due to impaired metabolism and elimination of tramadol [6]. The same can be said for patients taking CYP2D6 inhibitors, such as SSRIs. There are very few cases reported on opioid analgesics having contributed to the precipitation of SS. Given that the number of cases of the syndrome has gone up over the years and more patients are prescribed SSRIs, it is increasingly important for prescribers to be aware of this phenomenon and take into account the interactions between different medication classes.

## Conclusions

It is important to recognize serotonin syndrome in patients because many healthcare providers encounter this diagnosis during their careers. The clinical diagnosis of SS is challenging due to the syndrome having a wide range of presentations and it is often a diagnosis of exclusion. Our patient had a mild course and she had multiple factors in her history that might have influenced her presentation, including inflammatory illnesses as well as being on a SSRI and a serotonin antagonist and reuptake inhibitor, concomitantly with an opiate. The addition of tramadol was suspected to be the “tipping of the scale”. Our case is unique because as healthcare providers, we don’t often associate opioids with serotonin.
